# The Oak–Wood Extract Robuvit^®^ Improves Recovery and Oxidative Stress after Hysterectomy: A Randomized, Double-blind, Placebo-Controlled Pilot Study

**DOI:** 10.3390/nu12040913

**Published:** 2020-03-27

**Authors:** Vladimír Ferianec, Matej Fülöp, Miriam Ježovičová, Jana Radošinská, Marta Husseinová, Michaela Feriancová, Dominika Radošinská, Miroslav Barančík, Jana Muchová, Petra Hȍgger, Zdeňka Ďuračková

**Affiliations:** 1 Department of II. Gynecology and Obstetrics, Medical Faculty, Comenius University, 82606 Bratislava, Slovakia; ferianec@gmail.com (V.F.); michaelabart@gmail.com (M.F.); 2 Institute of Medical Chemistry, Biochemistry and Clinical Biochemistry, Medical Faculty, Comenius University, 81108 Bratislava, Slovakia; fulop.matej@gmail.com (M.F.); miriam.jezovicova@fmed.uniba.sk (M.J.); jana.muchova@fmed.uniba.sk (J.M.); 3 Institute of Physiology, Faculty of Medicine, Comenius University in Bratislava, 81108 Bratislava, Slovakia; jana.radosinska@fmed.uniba.sk (J.R.); marta.husseinova@fmed.uniba.sk (M.H.); 4 Center of Experimental Medicine, Institute for Heart Research, Slovak Academy of Sciences, 84104 Bratislava, Slovakia; miroslav.barancik@savba.sk; 5 Faculty of Natural Sciences, Comenius University in Bratislava, 84215 Bratislava, Slovakia; dominika.radosinska@fmed.uniba.sk; 6 Institut für Pharmazie und Lebensmittelchemie, Universität Würzburg, 97074 Würzburg, Germany; petra.hoegger@uni-wuerzburg.de; 7 Institute of Medical Chemistry, Biochemistry and Clinical Biochemistry, Faculty of Medicine, Comenius University, Sasinkova 2, 81108 Bratislava, Slovakia

**Keywords:** hysterectomy, Robuvit^®^, oak wood extract, post-operative recovery, oxidative stress, matrix metalloproteinases, complementary medicine

## Abstract

Hysterectomy has a variety of medical indications and improves pre-operative symptoms but might compromise the quality of life during recovery due to symptoms such as fatigue, headache, nausea, depression, or pain. The aim of the present study was to determine the effect of a standardized extract from French oak wood (*Quercus robur*) containing at least 40% polyphenols of the ellagitannins class, Robuvit^®^, on convalescence and oxidative stress of women after hysterectomy. Recovery status was monitored with the SF-36 questionnaire. The supplementation with Robuvit^®^ (300 mg/day) during 4 weeks significantly improved general and mental health, while under placebo some items significantly deteriorated. Oxidative stress and enhancement of MMP–9 activity was significantly reduced by Robuvit^®^ versus placebo. After 8 weeks of intervention, the patients’ condition improved independently of the intervention. Our results suggest that the use of Robuvit^®^ as a natural supplement relieves post-operative symptoms of patients after hysterectomy and reduces oxidative stress. The study was registered with ID ISRCTN 11457040 (13/09/2019).

## 1. Introduction

The surgical removal of the uterus, a hysterectomy, is one of the most common operative gynecological procedures. 

Total hysterectomy refers to the removal of the whole uterus including the cervix, subtotal hysterectomy refers to the removal of the uterus without the cervix. The latter is preferred by some surgeons in order to decrease the negative effect on pelvic floor and sexual functions, however, this has not been confirmed [1].

Indications for hysterectomy include menorrhagia as the most frequent indication in premenopausal women due to myomas or adenomyosis, pelvic pain due to endometriosis/adenomyosis after unsuccessful conservative treatment and uterine prolapse. Malignancy or postpartum hemorrhage account only for 10% of all hysterectomies [2]. 

In general, hysterectomy improves psychological and general wellbeing of the patients due to removal of the symptoms that led to the surgery. However, patients’ quality of life during postoperative recovery can be significantly impaired by symptoms such as fatigue, headache, nausea, depression, pain, wound infection and bleeding [3,4]. These symptoms may have an important social and economic impact [5].

So far, only a few studies have investigated the pathophysiology of the aforementioned postoperative symptoms. Some studies determined an association between fatigue, depression, anxiety, nausea and headache with oxidative stress [6,7,8,9].

During surgery, biological pathways leading to oxidative stress and the production of free radicals are activated [10,11]. The extent of oxidative stress depends on many factors such as the type, extent and time of surgery, operation technique, type of anaesthesia or perioperative analgesia [12,13,14,15,16,17,18,19].

Despite many studies on aspects of postoperative recovery, no consensus about a validated procedure to accelerate or assist recovery in this period exists [6].

**Figure 1:**
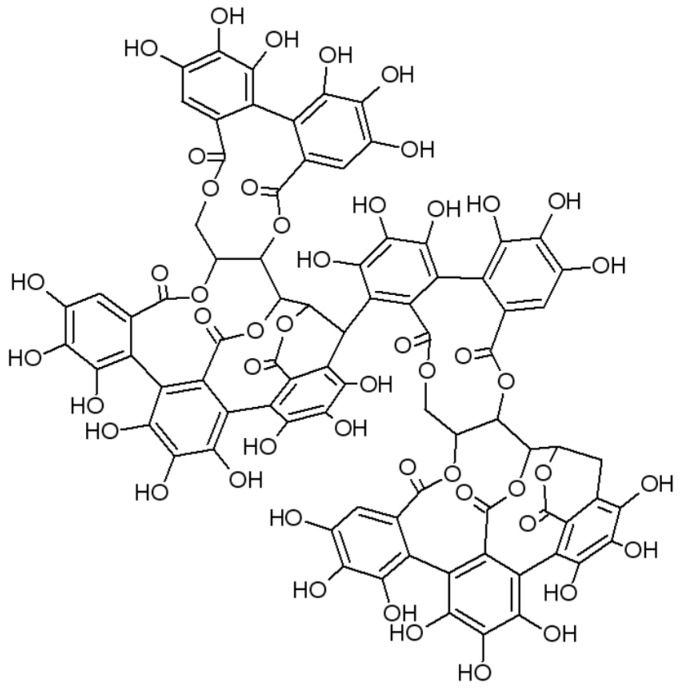
Chemical structure of roburin A.

Based on the reported positive effects of Robuvit^®^ on energy levels and mental health [7] and the outcomes of clinical studies related to the improvement of fatigue symptoms [8,9,10], burnout [11], recovery after sport [20,21] or fatigue in medical convalescence [22], all in conjunction with the reduction of oxidative stress, we aimed to investigate the influence of Robuvit on the recovery of women after hysterectomy. Robuvit^®^ (Horphag Research) (further referred to as Robuvit) is the proprietary water extract from the wood of French oak (*Quercus robur L.*) and contains at least 40% polyphenols such as ellagitannins. Roburins are ellagitannin dimers of which five structures have been reported so far, named A (Figure 1), B, C, D and E. Ellagitannin monomers are the stereoisomers vescalagin and castalagin [23,24]. In addition to roburins, Robuvit also contains gallic acid and ellagic acid [25,26].

Due to their high molecular weight, ellagitannins are not absorbed in the gastrointestinal tract, but are metabolized by gut bacteria yielding urolithins [27]. Urolithins A, B and C have been detected in plasma samples after the intake of Robuvit (25).

In addition to the antioxidant properties of ellagitannins, anti-carcinogenic [28,29], anti-inflammatory effects [30,31], antithrombotic [32] and antiplatelet properties [33] were described for ellagic acid. In our previous study, an increased total antioxidant capacity and a higher activity of antioxidative enzymes such as superoxide dismutase and glutathione peroxidase as well as decreased levels of advanced oxidation protein products and lipoperoxides were determined after Robuvit administration (300 mg/day) to adult volunteers [34]. Additionally, in a pilot study, positive effects of Robuvit on mental health and energy level in healthy volunteers have been detected [7]. Evidence from gene expression analysis suggested that metabolites in the serum of volunteers after the intake of Robuvit can be responsible for improving functioning of ribosomes [25]. In addition, it was shown that urolithin A induces mitophagy promoting the regeneration of mitochondria for an improved performance [35].

Matrix metalloproteinases (MMPs) are enzymes responsible for the cleavage of extracellular matrix. They are important for normal tissue remodelling, but also contribute to matrix degradation under various pathologies [36,37]. The MMP family comprises almost 30 members that are historically divided into groups mostly by their substrate preference with MMP–2 and MMP–9 being gelatinases [36,38]. MMPs play a significant role in inflammatory processes. Any surgery involves tissue injury and is thus an event resulting in the onset of an inflammatory response. The subsequent wound healing is a complex process involving inflammation, proliferation and remodelling. The review by Pilcher et al. [39], details the role of MMPs in cutaneous wound healing.

The primary outcome of the present study was to determine how Robuvit affected the mental and physical wellbeing of patients after vaginal hysterectomy followed by eight weeks of supplementation with Robuvit in a randomized, double-blind, placebo-controlled study. The secondary outcome was to determine the effect of Robuvit on oxidative stress and matrix metalloproteinase MMP–2 and MMP–9 levels and activities.

## 2. Material and Methods

**Subjects:** Seventy female patients (age 52.9 ± 9.4 years) with a medical indication for vaginal hysterectomy were recruited to participate in the study between February 2015 and June 2018. All the patients underwent hysterectomy for benign indications such as meno–/metrorrhagia or uterine prolapse. Preoperatively, the patients did not take any analgesics or other drugs related to the symptomatology. Patients were registered at the Department of II. Gynecology and Obstetrics, Medical Faculty, Comenius University, Bratislava, Slovakia. 

*Inclusion criteria:*

1. Age above 18 years.
2. Medical indication for hysterectomy for benign indications such as meno/metrorrhagia or uterine prolapse.
3. Signed informed consent.

*Exclusion criteria:*

1. Being enrolled in another study.
2. Malignant disease, severe systemic disease, chronic autoimmune diseases.
3. Significant psychiatric disorders or receiving antipsychotic drug.
4. Preoperative intake of analgesics or other drugs related to the symptomatology.

Since the present study was designed as an investigational pilot trial, a patient sample size was not calculated.

**Randomisation and blinding**: Patients who underwent preoperative gynecological examination at the gynecological clinic and met the inclusion criteria were informed about the study and the possibility of enrolment. They were acquainted with the study protocol and answered the study questions. Patients participating in the trial were assigned a sequential trial number (patient number). The list of trial numbers was linked to a computer-generated randomization list by StatsDirect program (randomized with seed: 57826). Capsules of Robuvit and placebo, identical in size, weight, colour and appearance, were delivered in boxes with code numbers to the Institute of Medical Chemistry, Biochemistry and Clinical Biochemistry of the Comenius University in Bratislava. The principal investigator, retaining the code, transferred the boxes to a technician of the same institute. The technician distributed the capsules of Robuvit or placebo from the coded boxes to the sample boxes for patients according to the randomisation scheme. The sample boxes for patients, marked with the sample number, were transferred to the Department of Gynecology and Obstetrics of the Medical Faculty. The technicians, the patients, the physicians and the statistician, evaluating the results of the study, were blinded until the end of the statistical evaluation.

**Intervention:** After vaginal hysterectomy, capsules were dispensed to patients starting on the third day postoperatively. Patients received either 100 mg Robuvit three times daily (300 mg/day) (group with Robuvit) or three capsules placebo (group with placebo) for 8 weeks. Placebo capsules were of the same shape and appearance as capsules containing Robuvit. Robuvit^®^ is a registered trademark of Horphag Research. Robuvit and placebo capsules were provided by Horphag Research.

Patients were instructed to follow a standard diet, not to take antioxidant and polyphenol supplements.

Clinical examination was performed before the intervention (week 0), after 4 and 8 weeks of Robuvit/placebo intervention. Patients were contacted by a doctor every week by phone and asked about compliance and possible problems. Fasting venous blood was collected before surgery (week 0) and 4 and 8 weeks after start of Robuvit or placebo administration.

**Figure 2:**
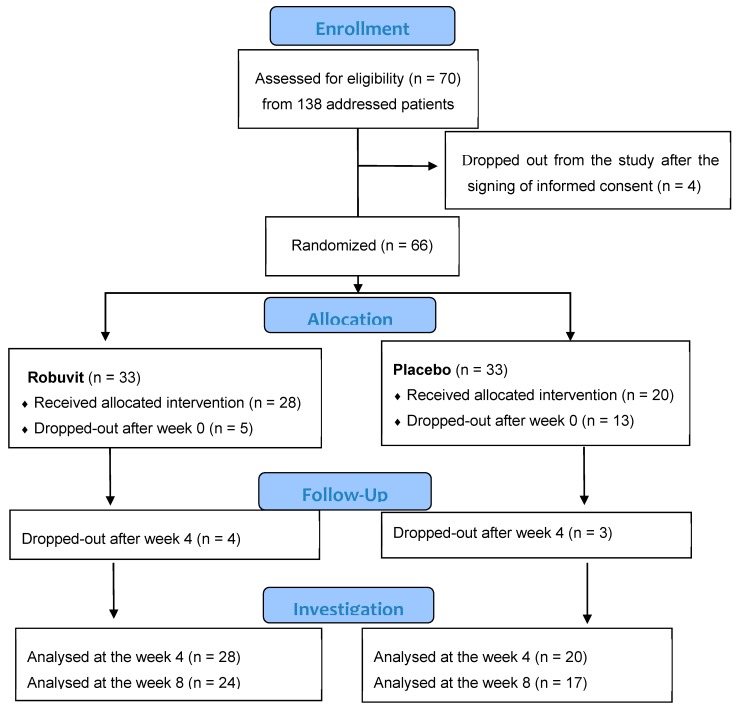
CONSORT (Consolidated Standards of Reporting Trials) flow diagram of the study.

Four patients dropped out after signing informed consent. Sixty-six patients were included in this double-blind, randomized, placebo-controlled study and randomized into the active (33) and placebo groups (33). Five patients from the Robuvit and 13 patients from the placebo group dropped out shortly after surgery. Dropped out patients did not report any medical, but social or psychological reasons. The remaining patients completing the 4-week intervention (Robuvit group 28, placebo 20) and the 8-week intervention (Robuvit 24, placebo 17) were evaluated (Figure 2). 

**Monitoring of post-operative recovery (primary outcome):** Patients post-operational recovery was evaluated with the standardized and validated questionnaire SF-36 for health survey. Answers of patients were scored at baseline and after 4 and 8 weeks of intervention. All questionnaires underwent blinded analysis by a physician according to Hays et al. [40].

**Biochemical analyses (secondary outcome):** Fasting venous blood was collected before surgery (week 0) and after 4 and 8 weeks after the start of Robuvit/placebo administration (week 4, week 8). 

Blood plasma was obtained by centrifugation (10 min at 1200× *g*) using EDTA as an anticoagulant. Serum was obtained by the standard procedure for blood samples by centrifugation in the absence of an anticoagulant. Blood plasma and serum samples were aliquoted and kept at −80 °C until analyses.

Basic biochemical parameters (glucose, creatinine, uric acid, total proteins, triacylglycerols, total cholesterol, LDL- and HDL-cholesterol and high sensitivity C-reactive protein (hsCRP) were determined by standard biochemical procedures in Medirex servis, s.r.o. Bratislava. 

The antioxidant capacity of plasma was determined by the TEAC method (trolox equivalent antioxidant capacity) on the Hitachi 911 automated analyser using the Randox set (United Kingdom). Antioxidant activity was expressed in nmol trolox/mL of plasma [41].

Lipid hydroperoxides (LPx) in blood serum were determined according to El–Saadani et al. [42], advanced oxidation protein products (AOPP) according to the modified Witko–Sarsat et al. [43].

The gelatinolytic activities of metalloproteinases (MMPs) in plasma were analyzed by zymography in 10% polyacrylamide gels containing gelatin as a substrate as described previously [44]. MMP activities were visualized as transparent bands against a dark-blue background and quantified by ImageJ software (National Institutes of Health, Bethesda, USA). MMP–9 and MMP–2 proteolytic activities were identified by determination of the molecular weight of the bands in the gels. The change in MMPs activity was expressed as % change compared to baseline activity (week 0), which was preset to 100%.

For Western blot analysis, after an electrophoretic separation, the proteins were transferred onto a nitrocellulose membrane as described previously [45]. The quality of the transfer was verified by Ponceau S staining of the nitrocellulose membranes after transfer. A specific anti-MMP–9 antibody (H–129, Santa Cruz Biotechnology, dilution 1:333) was used as the primary antibody. Peroxidase-labeled anti-rabbit immunoglobulin (7074S, Cell Signaling Technology, dilution 1:2000) was used as the secondary antibody. The bound antibodies were detected by the enhanced chemiluminescence detection method. The change in MMPs level was expressed as % change compared to baseline level (i.e., before the surgery) which was preset to 100%.

## 3. Statistics

Normality of data was determined with the Shapiro–Wilk test. The data of the groups at the individual weeks of investigation were compared relative to baseline values using the paired t–test (two sided) or Wilcoxon test, depending on the data distribution. The data difference between the Robuvit and the placebo groups at the given weeks was analysed by the Mann–Whittney test. Friedman’s test was used for all pairwise comparisons (Conover) for analysing differences in treatments (Robuvit/placebo). Pearson correlation was calculated to determine correlations between parametric data and Spearman correlation between non-parametric data. 

Statistical analyses were performed with StatsDirect^®^ 3.1.22 (StatsDirect Sales, Sale, Cheshire, M33 3UY, UK) and IBM SPSS Statistics 23. GraphPad Prism 8 was used to prepare the figure. *p* < 0.05 was considered as significant in all statistical analyses. 

## 4. Results

Basic demographic data of patients revealed no statistical difference between the treatment groups (Table 1).

Robuvit was well tolerated by all patients. No unwanted effects were observed during the study. 

**Figure 3:**
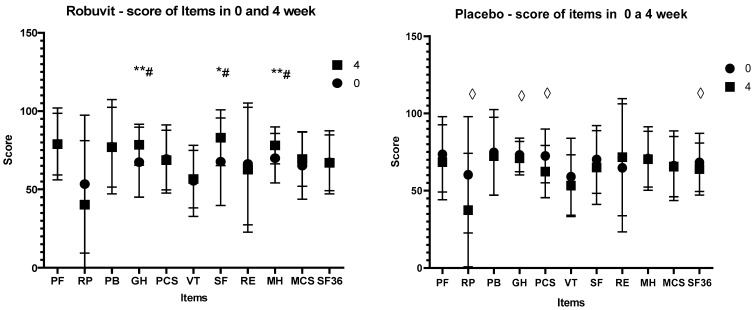
Changes in scores of individual health items after 4–week Robuvit and placebo administration (numbers of analysed data in both groups are given in Flow diagram). R—Robuvit, Pl—placebo, PF—physical functioning, RP—role limitations due to physical health, PB—pain, GH—general health, PCS—physical Component Summary, VT—vitality, SF—social functioning, RE—role limitations due to emotional problems, MH—mental health, MCS—mental component Summary, SF–36, whole physical and mental health. * *p <* 0.05 for positive difference in Robuvit group between week 4 and week 0, ** *p* < 0.01 for positive difference in Robuvit group between week 4 and week 0, ◊ *p* < 0.05 for negative difference in placebo group between week 4 and week 0, # *p* < 0.05 for difference between Robuvit and placebo groups.

Recovery state was monitored with the SF–36 questionnaire for the health survey, in which higher scores indicate better function/health. The score values of the SF–36 items at the baseline (before the intervention, week 0) between the Robuvit and placebo groups were not significantly different. However, after 4 weeks of Robuvit intervention, there was a significant difference between the Robuvit group and placebo in the general health (GH; 79.59 vs. 71.04, *p* = 0.016), social functioning (SF; 79.54 vs. 65, *p* = 0.031) and mental health (MH) score (78.62 vs. 72.4, *p* = 0.019) (Figure 3).

The treatment effect in the Robuvit group examined by the Friedman‘s test in SF–36 questionnaire was significant (*p* = 0.028). In contrast, significance of treatment effect was not confirmed for the placebo (*p* = 0.101). 

In contrast, negative scores were recorded in the placebo group for role limitations due to physical health (RP) (*p* = 0.014), for general health (GH) (*p* = 0.042), for physical component summary (PCS) (*p* = 0.014) and whole physical and mental health (SF–36) (*p* = 0.043) (Figure 3, Supplement S1).

Changes in scores of individual health items after Robuvit and placebo after 8-week administration exceeded the baseline value score of all items in both groups (Supplement S1).

Biochemical parameters (glucose, creatinine, uric acid, triacylglycerols) were within the range of physiological values before surgery. However, before the surgery total cholesterol and LDL cholesterol were slightly elevated above the physiological range (5.35 and 3.84 mmol/L, respectively). Total protein level (TP) increased after 8 weeks of Robuvit intake from 66.17 to 72.19 g/L (*p* = 0.003) but remained within the range of the physiological values. In the placebo group, total protein level did not change significantly from 67.9 to 69.16 g/L (*p* = 0.237). hsCRP was within the reference range at the beginning as well as after 8 weeks of intervention.

Markers of oxidative stress and activities of MMP–9 and MMP–2 before and after 4 and 8 weeks intake of Robuvit or placebo are summarised in Table 2. As baseline values of MMP–9 and MMP–2 varied among the patients, we set each basic activity (week 0) to 100%. After 4 and 8 weeks, measured activities were adjusted to corresponding baseline values in order to reveal changes in respective MMP activities.

Levels of oxidative stress markers (AOPP, lipoperoxides and TEAC), as well as activity of MMP–9 were significantly different between Robuvit and placebo groups after the 4 weeks of intervention, indicating that Robuvit decreased oxidative stress. After 8 weeks, MMP–9 activity was not different anymore between the Robuvit and placebo groups. MMP–2 activities were higher both after 4 and 8 weeks of intervention in both groups (Robuvit and placebo) compared to baseline. However, there was no difference between Robuvit and the placebo group (Table 2).

The difference in MMP–9 activities between the Robuvit and placebo groups after 4 weeks was accompanied by unchanged protein concentrations of this enzyme in plasma samples (median 101.9% in placebo, 105.5% in Robuvit, *p* = 0.83).

Use of analgesics during the recovery period underlined these findings since intake of analgesics was considerably lower in the Robuvit group (8/28) as compared to placebo (10/20).

The correlation analysis between oxidative stress markers, MMP activities and significantly altered parameters in the SF–36 questionnaire revealed no correlation between MMP levels and significantly changed items of the SF–36 questionnaire after 4-weeks (GH, SF, MH). However, higher MMP–9 activity correlated with lower antioxidant capacity (Spearman’s rank correlation coefficient (Rho) = −0.473043, 95% CI for rho (Fisher’s Z transformed) = −0.735995 to −0.086071, Two sided *p* = 0.0207), unlike the placebo, where this correlation was not significant (*p* = 0.183).

## 5. Discussion

The improvement of general and mental health under Robuvit, in contrast to the post-operative worsening of general health under the placebo, suggested that the use of Robuvit improved post-operative recovery and reconvalescence within the four weeks following surgery, leading to significant differences in scores for general and mental health and social functioning in favour of Robuvit.

The fact that scores of all items in the SF–36 questionnaire where higher compared to baseline values after 8 weeks for Robuvit as well as for placebo, indicated that patients experienced a full recovery, a complete convalescence and improved well-being due to the of elimination of pre- and post-operative symptoms after 8 weeks.

The results of the present study are in accordance with results of our preceding study, demonstrating an improvement of energy metabolism and mental health of healthy adults by supplementation with Robuvit [7]. In patients recovering from hysterectomy, significant improvements of general health (+16.5%), social functioning (+22.5%) and mental health (+11.7%) relative to baseline were observed after 4 weeks. In contrast, the patients in the placebo group reported a significant worsening of role of limitations due to physical health (−37.8%), general health (−3%), physical component summary (−14%) and general health (SF–36; −6.4%). The differences between the Robuvit and placebo group in general health, social functioning and mental health indicated a highly significant improvement of mental and physical well-being by Robuvit supplementation. Increasing scores of all evaluated items after 8 weeks of supplementation over baseline in both groups indicated full recovery of patients.

The use of Robuvit over 8 weeks of hysterectomy did not affect the basic biochemical parameters (glucose, creatinine, uric acid, triacylglycerols, total- LDL- and HDL-cholesterol, hsCRP) in patients, with the exception of the total protein levels, that were significantly increased in the Robuvit group. Natella et al. [25] found that Robuvit metabolites present in the serum of volunteers who took Robuvit for 5 days affected ribosomes, cell cycle and spliceosome pathways. Based on those results it could be speculated that Robuvit improved the functioning of cellular ribosomes and enhanced protein synthesis, thereby supporting recovery after surgery [46,47,48].

Our results confirmed the antioxidant potency of Robuvit which prevented elevation of AOPP levels in patients after hysterectomy in contrast to placebo, where the levels of the mentioned marker were significantly increased. Lipoperoxides decreased in the Robuvit group, but not in the placebo group, after 4 weeks of treatment. Antioxidant capacity increased in the Robuvit group, but not under placebo after the 4 and 8 weeks of intervention. This is consistent with the findings of Horváthová et al. [34], Ippolito et al. [22] and Belcaro et al. [49].

Various research groups described increased activities of MMP–9 in patients after surgery [50,51,52]. However, the increase of MMP–9 activities in our patients was significantly lower after four weeks of intervention with Robuvit compared to the placebo (*p* = 0.006). As we did not simultaneously observe a difference in MMP–9 protein concentrations between the placebo and Robuvit groups, the decrease in MMP–9 activity in the Robuvit group was not based on decreased protein synthesis. 

It has been described that MMP activation may be a consequence of posttranslational modification induced by oxidizing agents [53]. An inhibition of this activation by Robuvit would be consistent with the antioxidant action documented in the present study. A similar effect on the activation of both MMP–9 and MMP–2 proteins was found after application of quercetin in mice with abdominal aortic aneurysms [54]. Another possible explanation for the lower MMP activity would be that constituents or metabolites of Robuvit acted as direct inhibitors of MMP.

It has been discussed that MMP–9 inhibition modulates inflammatory reactions, resulting in faster neovascularization of connective tissues in an animal hernia model [55]. MMP inhibition with doxycycline was associated with improved hernia repair, concomitant reduction of MMP levels and increased collagen 1 deposition [56]. Moreover, curcumin application resulted in faster wound closure compared to a control group, downregulation of MMP–9 expression and increased collagen production [57]. MMP–2 and MMP–9 were suggested as effective biomarkers for wound status [58].

We did not observe a direct association between oxidative stress and MMP activities, except for the negative correlation between MMP–9 activity and total antioxidant status in the Robuvit group after 4 weeks. Fu et al. [59] found that pro-inflammatory cytokines increase MMP–9 expression which may contribute to the association of increased inflammation with increased oxidative stress and increased MMP–9 activity. Suppressing oxidative stress with Robuvit can at least partially contribute to MMP–9 inhibition.

A limitation of the present study was the use of one questionnaire form for assessment of the physical and mental condition of patients during convalescence in the home environment. Another limitation was the low number of patients being evaluated in this pilot trial. Hence, the results should be confirmed in a bigger study. Another limitation is that we did not control the dietary habits and treatment of patients after hysterectomy with antibiotics. This could influence the formation of different metabolites of Robuvit with different biological activities by different bacterial flora in individual patients [60]. To conclude, supplementation with Robuvit for 4 weeks improved convalescence of patients following hysterectomy, probably through its antioxidant activity as well as by the inhibiting of MMP–9 activity. An increased synthesis rate of proteins might have played a role in recovery as well. Further studies are needed in order to evaluate whether Robuvit should be generally recommended for improvement of post-operative reconvalescence.

## Figures and Tables

**Table 1:** Basic demographic data of patients.

Group	Robuvit	Placebo	p
n	33	33	
Age in years ± SD	51.0 ± 9.1	50.3 ± 9.0	0.135
BMI ± SD	27.3 ± 5.3	27.1 ± 4.9	0.563

BMI—body mass index (weight (kg)/height (m^2^)), p—significance, n—number of patients, SD—standard deviation.

**Table 2:** Markers of oxidative stress and MMP–9 and MMP–2 before and after 4 and 8 weeks of intervention with Robuvit or placebo.

	Robuvit			Placebo			p	p
Week of Intervention	0	4	8	0	4	8	R vs. Pl at W4	R vs. Pl at W8
AOPP (µmol/L)	64.0 ± 28.7	62.0 ± 22.7	68.4 ± 22.9	65.4 ± 25.8	100.7 ± 42.3	102.5 ± 41.1		
n	28	24	24	27	20	18		
p W vs. W0		0.663	0.961		**0.010**	**0.02**	**<0.001**	**<0.01**
Lipoperoxides (nmol/mL)	36.2 ± 10.4	30.9 ± 9.8	31.4 ± 9.5	36.3 ± 10.3	40.2 ± 8.5	39.1 ± 8.02		
n	28	24	24	27	20	16		
p W vs. W0		**<0.001**	**<0.001**		0.622	0.597	**0.030**	**0.017**
TEAC(nmol/mL)	3.27 ± 0.41	3.55 ± 0.37	3.89 ± 0.43	3.18 ± 0.47	3.11 ± 0.31	3.45 ± 0.53		
n	26	26	26	20	20	18		
p W vs. W0		**0.0164**	**<0.001**		0.202	0.145	**0.001**	**0.015**
MMP–9 (% of W0)	100	107.9 ± 20.1	106.8 ± 45.9	100	136.7 ± 39.9	111.3 ± 49.4		
n	28	28	27	21	20	19		
p W vs. W0		0.086	0.546		**0.0012**	0.490	**0.0064**	0.912
MMP–2 (% of W0)	100	133.5 ± 62.8	135.8 ± 62.1	100	130.3 ± 27.5	129.7 ± 34.6		
n	27	27	27	19	19	19		
p W vs. W0		**0.005**	**0.007**		**0.0002**	**0.0017**	0.341	0.611

AOPP—Advanced oxidation protein products; TEAC—trolox equivalent antioxidant capacity; MMP—matrix metalloproteinase; p—statistical significance; W—week; n—number of samples; vs.—versus.

## Supplementary Materials

The following are available online at https://www.mdpi.com/2072-6643/12/4/913/s1, Supplement S1: Score values for each item in the Robuvit and placebo groups at baseline and at weeks 4 and 8 of supplementation.
