# Supplementary material for: The Oak–Wood Extract Robuvit® Improves Recovery and Oxidative Stress after Hysterectomy: A Randomized, Double-blind, Placebo-Controlled Pilot Study
Source: Nutrients. 2020 Mar 27;12(4):913. doi: 10.3390/nu12040913 (PMC7230691; doi:10.3390/nu12040913)
Supplement: Supplementary file 1 [file nutrients-12-00913-s001.pdf]

Suplement S1. Score values for each item in the Robuvit and placebo groups at baseline and at weeks 4 and 8 of supplementation.

	Score (average) – Robuvit					Score (average) – placebo					P Robuvit vs. placebo		
Items	W0	W4	P W4 vs W0	W8	P W8 vs W0	W0	W4	P W4 vs W0	W8	P W8 vs W0	W0	W4	W8
n	32	28		24		23	23		23				
PF	79.1	78.9	0.911	86.3	**0,001**	73.5	68.5	0.169	89.7	0,107	0, 542	0,074	0,998
RP	53.4	40.3	0.632	81.3	**0,001**	60.3	37.5	**0.014**	82.4	0,382	0,588	0,816	0,856
PB	77.3	76.9	0.976	86.8	**0,083**	74.8	72.4	0.223	92.1	0,129	0,965	0,649	0,605
GH	67.4	78.5	**0.001**	80.1	**0,028**	73.2	71.0	**0.042**	74.7	0,758	0,237	**0,017**	0,194
PCS	69.4	68.7	0.775	83.6	**0,001**	72.5	62.4	**0.014**	84.7	**0,019**	0,700	0,091	0,963
VT	55.5	56.6	0.356	67.1	**0,011**	59.1	53.3	0.413	67.6	0,271	0,555	0,585	0,879
SF	67.7	83.0	**0.016**	87.5	**0,001**	70.2	65.0	0.131	77.2	0,978	0,633	**0,031**	**0,002**
RE	66.3	62.6	0.999	81.9	**0,031**	64.8	71.7	0.844	90.2	0,086	0,847	0,722	0,421
MH	69.9	78.1	**0.003**	79.5	**0,019**	70.8	70.4	0.389	82.6	0,138	0,262	**0,019**	0,273
MCS	65.2	70.1	0.069	79.0	**0,002**	66.2	65.1	0.279	79.4	0,117	0,732	0,301	0,860
SF36	67.3	67.0	0.691	81.3	**0,001**	68.3	64.0	**0.042**	82.1	**0,034**	0,614	0,608	0,642

PF - physical functioning, RP - role limitations due to physical health, PB - pain, GH - general health, PCS - physical Component Summary, VT - vitality, SF - social functioning, RE - role limitations due to emotional problems, MH - mental health, MCS - mental component Summary, SF36 - whole physical and mental health W – week, P – significance, vs - versus
